# Pneumonia in common variable immunodeficiency after change in environment

**DOI:** 10.1186/1939-4551-8-S1-A59

**Published:** 2015-04-08

**Authors:** Guilherme Guimarães, Ana Paula Kazue Beppu, Julia Warchavchik Melardi, Verônica Reche Rodrigues Gaudino, Marina Colella Dos Santos, Maria Da Conceição Santos De Menezes, Wilma Carvalho Neves Forte

**Affiliations:** 1 Faculty of Medical Sciences of Santa Casa De São Paulo, Brazil

## Background

Common Variable Immunodeficiency (CVID) is a Primary Immunodeficiency characterized by an association of IgG deficiency with IgA and/or IgM deficiency, and a decrease in the function of specific antibodies, discarding other causes of hipogamaglobulinemia. The recurrent pneumonia are among the main clinical manifestations of CVID,which appear between 20 and 40 years of age in most cases. The aim of this study was to analyse the possible relationship between environmental changes and the onset of pneumonia in patients with primary CVID.

## Methods

A prospective cross-sectional study was conducted after approval by the Institutional Review Board and written consent of the patients, as protocol number 16622913.5.0000.5479. Eighteen patients with CVID regularly followed in a specialized sector tertiary hospital were studied for one year (March 2013 to March 2014). Inclusion criteria were a previous diagnosis of CVID with a positive history of recurrent pneumonia. All received monthly replacement with human immunoglobulin. We investigated the clinical characteristics of the onset of pulmonary symptoms by standard questionnaire for all.

## Results

Among the patients studied, 10 were male and 8 female, with mean age of 23 years old (6 to 64 y). The median age for the onset of recurrent pneumonies was 6,5 years (3months to 32y). The mean age of CIVD diagnostic was 11 years (5 to 59 y). The time period from the onset of first pneumonia to the CVID diagnostic was 5,6 years. Five of 18 CVID patients have the positive relation between onset of clinical recurrent pulmonary infections after environmental changes.

## Conclusions

We observed that 28% of patients with CVID studied began presenting pneumonia after changes in the physical environment, especially after living with a greater number of individuals. We believe it is important hypothesis CVID in patients with recurrent pneumonia after changes in the physical environment. It is possible that part of the bimodal age distribution CVID diagnostic result of changes in the physical environment. More studies are needed in order to know the cause of the clinical manifestations are delayed at CVID.

